# The indexing ambiguity in serial femtosecond crystallography (SFX) resolved using an expectation maximization algorithm

**DOI:** 10.1107/S2052252514020314

**Published:** 2014-09-23

**Authors:** Haiguang Liu, John C.H. Spence

**Affiliations:** a Physics Department, Arizona State University, Tempe, AZ 85282, United States; b Beijng Computational Science Research Center, 3 Heqing Rd, Haidian, Beijing 100084, People’s Republic of China

**Keywords:** indexing ambiguity, serial femto­second crystallography (SFX), XFELs, protein crystallography, expectation maximization algorithm

## Abstract

Crystallographic auto-indexing algorithms provide crystal orientations and unit-cell parameters and assign Miller indices based on the geometric relations between the Bragg peaks observed in diffraction patterns. However, if the Bravais symmetry is higher than the space-group symmetry, there will be multiple indexing options that are geometrically equivalent, and hence many ways to merge diffraction intensities from protein nanocrystals. Structure factor magnitudes from full reflections are required to resolve this ambiguity but only partial reflections are available from each XFEL shot, which must be merged to obtain full reflections from these ‘stills’. To resolve this chicken-and-egg problem, an expectation maximization algorithm is described that iteratively constructs a model from the intensities recorded in the diffraction patterns as the indexing ambiguity is being resolved. The reconstructed model is then used to guide the resolution of the indexing ambiguity as feedback for the next iteration. Using both simulated and experimental data collected at an X-ray laser for photosystem I in the *P*6_3_ space group (which supports a merohedral twinning indexing ambiguity), the method is validated.

## Abstract 2

An expectation maximization algorithm is implemented to resolve the indexing ambiguity which arises when merging data from many crystals in protein crystallography, especially in cases where partial reflections are recorded in serial femtosecond crystallography (SFX) at XFELs.

## 1. Introduction   

An X-ray free electron laser (XFEL) generates brief intense X-ray pulses of femtosecond duration, allowing structure determination of sub-micron crystals. This technique, known as serial femtosecond crystallography (SFX), has provided atomic resolution structures for proteins (Barends *et al.*, 2014 ▶; Boutet *et al.*, 2012 ▶; Chapman *et al.*, 2011 ▶; Liu *et al.*, 2013 ▶; Redecke *et al.*, 2013 ▶). At the same time, SFX has created many challenges for data analysis, since patterns from thousands of nanocrystals of different sizes and orientations must be merged after indexing, and also because of the large shot-to-shot variations in pulse intensity. Here, we discuss one of the most important issues in SFX, namely the indexing ambiguity which occurs when the Bravais symmetry is higher than the space-group symmetry. In its simplest form, the indexing ambiguity arises when a correctly indexed crystal might also be indexed as its twin. We do not suggest here that the nanocrystals themselves are twinned, only that data from different single crystals may be accidently merged in twin-related orientations. Thus, merged data from patterns subject to a twinning index ambiguity are similar to those from physically twinned crystals. The process of resolving this ambiguity has thus been referred to as detwinning.

Auto-indexing software, including the widely used *MOSFLM* (Leslie, 1999 ▶), *DIRAX* (Duisenberg, 1992 ▶) and *LABELIT* (Sauter *et al.*, 2004 ▶) programs, are capable of determining both the unit-cell parameters and the orientation of a crystal, based on Bragg spot locations in a diffraction pattern. Indexing ambiguities are not a severe problem in conventional crystallography, because (i) a series (or even a full set) of diffraction patterns with known relative rotational relations are measured from each crystal; (ii) the reflections measured at each Bragg spot are narrowly distributed and represent the full diffraction intensities, especially if the oscillation or rotation technique is applied during each exposure. In SFX, a crystal is completely destroyed immediately after interacting with the X-ray laser pulse. The rotation of a nanocrystal can be ignored during the femtosecond exposure, so that the recorded intensity covers only a small fraction of the angular width of a reflection generated by mosaicity. This fraction is set by the range of wavelengths within each pulse (typically 0.1% monochromaticity), so that full structure factors cannot be extracted from a single diffraction pattern. The orientational relationship between diffraction patterns is unknown because the crystals are oriented randomly from shot to shot, and the auto-indexing methods cannot distinguish between, for example, twin-related indexing schemes based on Bragg spot locations alone. In such a case, if data were merged based on indexing results, one would expect half the data to be merged incorrectly by chance for the cases where two indexing modes are equivalent, resulting in a merged data set that appears to be from physically twinned crystals. On the other hand, the intensities of full reflections at Bragg spots can provide the extra information needed to resolve this ambiguity.

In SFX indexing a second challenge arises which is that, for mosaic crystals, the measured snapshot XFEL Bragg spots are only partial reflections. This is a result of the collimation and monochromaticity of the X-ray laser, which samples only a fraction of the full angular width of each reflection, which is broadened by mosaicity (Hattne *et al.*, 2014 ▶). Mosaicity models differ according to the type of crystal, and a model based, for example, on continuous elastic deformation may apply in some cases [see Snell *et al.* (2003 ▶) for a review].

Partiality has been defined in several ways, often very loosely as the angle by which a given diffracted beam misses the exact Bragg condition. More precisely, we will define the partiality of a reflection as the fraction of a full reflection that is recorded in one experimental pattern, with a maximum value of unity corresponding to a full reflection. A different situation arises for the second case of the smallest nanocrystals which consist of a single mosaic block (with possible elastic deformation), smaller than the width of the coherent beam, where the full Fourier transform of the external shape of the nanocrystal (the so-called shape transform) is laid down around every reciprocal lattice point. Interference fringes are then seen running between Bragg spots (Chapman *et al.*, 2011 ▶; Spence *et al.*, 2011 ▶) and the angular distribution of scattering around the Bragg condition can no longer be modelled as a smooth narrow peak. We may also have a third case, when using the smallest sub-micron diameter XFEL beams, where the beam size may be smaller than one ‘mosaic block’ or isolated nanocrystal. This case of coherent convergent-beam diffraction is treated by Spence *et al.* (2014 ▶) and will not be considered here. The fourth case of a large perfect crystal (such as a semiconductor silicon wafer) in which dynamic scattering is important will also not be considered here, since it does not occur in protein crystallography.

Since the intensity variation due to partiality variation may be much greater than that due to changes in the choice of indexing scheme, one has a chicken-and-egg problem, where full reflections are needed to resolve the indexing ambiguity, but these cannot be obtained until indexing is correctly assigned to allow data merging. The method of partial reflection analysis was developed by Rossmann & Erickson (1983 ▶) [see also Rossmann *et al.* (1979 ▶)] and has been used as a form of post-refinement ever since, in conjunction with modern goniometer-based data collection at synchrotrons. Recently, Brehm & Diederichs (2014 ▶) developed algorithms (BD algorithms) to resolve the indexing ambiguity by clustering the patterns based on pairwise similarities, measured using Pearson’s correlation coefficient. Here, we describe an expectation maximization (EM) algorithm to establish the relation between each diffraction pattern and iteratively construct the full correctly indexed three-dimensional diffraction volume. The important difference between the BD and EM algorithms lies in their evaluation functions. EM does not use the pairwise relationship between two sets of partial reflections (corresponding to the information recorded in two diffraction patterns). Rather, it utilizes the relationship between reflections from any pattern and a merged full reflection model, which is built up iteratively using the EM algorithm. This repeated comparison with an iteratively improved model greatly reduces the time to convergence and increases the accuracy of the method. We have implemented this algorithm within the framework of *CrystFEL* (White *et al.*, 2012 ▶) and tested it using both simulated patterns and experimental patterns from photosystem I crystals.

## 2. Method   

### 2.1. Existing methods and the Pearson correlation   

For two diffraction patterns, Pearson’s correlation coefficient is defined as (Brehm & Diederichs, 2014 ▶)
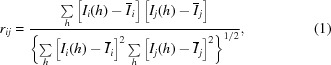
where {*I_i_*} and {*I_j_*} are the intensities measured from the two patterns *i* and *j*, and {*h*} are the indices for the common reflections. 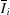 and 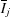 are the mean intensities calculated using common reflections from the two patterns. Clustering algorithms were then devised to group the patterns into the correct classes to assign indices consistently. Specifically, Brehm and Diederichs demonstrated success in classifying diffraction patterns to indexing modes by mapping the patterns into hyperspace, using the BD algorithm. For any pair of diffraction patterns, two quantities are defined to describe their relationship. One is the Pearson distance, which is defined as (1.0 − *r*_*ij*_), where *r*_*ij*_ is the Pearson correlation efficient [see equation (1)]. The second relation is the Euclidean distance (or the angle) between these two patterns in the hyperspace where the diffraction patterns are embedded and represented as points (or vectors from the origin, when considering the angles between patterns). In the BD algorithm, the difference between the Pearson distance and the Euclidean distance (or angles) is minimized. This procedure eventually maps closely related patterns to closer locations in hyperspace, thereby separating the indexing modes.

Pairwise correlations between diffraction patterns that record only partial intensities may not be accurate enough to discriminate one indexing mode from the alternatives (especially for the smallest nanocrystals), yet the success of the BD algorithm indicates the feasibility of clustering by exploiting pairwise correlations between patterns. It is also found that the average Pearson correlation between one pattern and all other patterns can be used to resolve the indexing ambiguity, and eventually leads to indexing in a consistent manner. The method described in the following also uses Pearson’s correlation coefficient to assign indices such that the patterns are indexed consistently. Considering that individual patterns consist of a set of ‘partial reflections’, we pursue the idea that comparison between partial reflections recorded in individual patterns against a model with full reflections should yield more reliable results. Therefore, we devised a Pearson correlation between a diffraction pattern *i* and a model consisting of full reflections, {*I*_full_}, defined as follows:
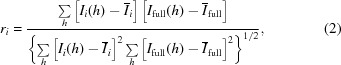
where *r*_*i*_ is the correlation coefficient and all other symbols are the same as in equation (1). We now describe how to construct the full reflection data set iteratively from the partial reflection data set, using an expectation maximization approach.

### 2.2. Expectation maximization algorithm and full reflection construction   

Full intensities in three-dimensional reciprocal space can be computed from an atomic model for a known structure. These {*I*_full_} can then be used as a reference to recover orientation information for each diffraction pattern. In practice, {*I*_full_} is often not known for a macromolecule, and it can only be determined by merging diffraction patterns after recovering the correct orientations or indexing modes. This comes back to our ‘chicken-and-egg’ problem, *i.e.* the ‘correct indexing and full reflection data set’, which is solved using the expectation maximization (EM) method. The EM approach has been applied to a related problem: determining the orientation of scattering patterns from single-particle experiments, and merging the scattered intensity into the three-dimensional scattering intensity volume. We have adapted the implementation of Tegze & Bortel (2012 ▶), which utilizes the Pearson correlation coefficients to assign orientations. Details of the EM method and its implementation can be found elsewhere (Loh & Elser, 2009 ▶; Tegze & Bortel, 2012 ▶). In brief, the algorithm works as follows.

**Figure 1:**
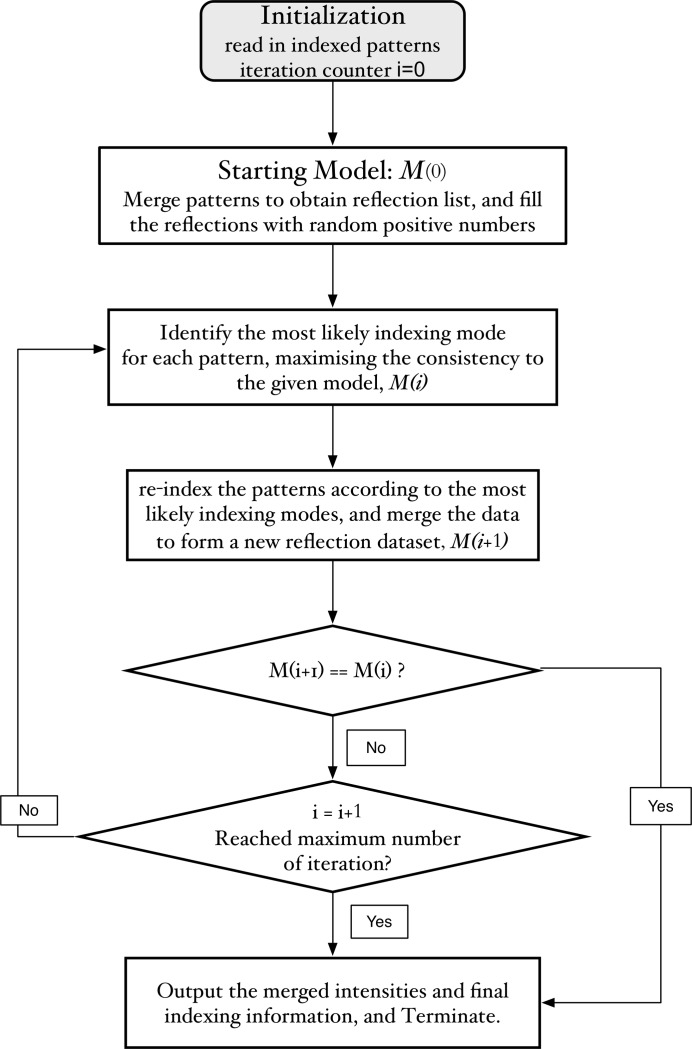
Flow chart for the expectation maximization algorithm.

At the *n*th iteration, each two-dimensional experimental diffraction pattern (consisting of partial reflections) is compared with a three-dimensional model of the full reflection intensities on the reciprocal lattice. Correlation coefficients {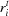} are computed between each pattern *i* and the model for each indexing mode *t*, where *t* enumerates all possible indexing possibilities (two, for the case of a twin-like ambiguity). The full set of two-dimensional patterns are then merged with each other into a three-dimensional diffraction volume, based on the indexing mode which shows the highest correlation with the current model. This newly merged model is then used as the reference for the next iteration, in which this process is repeated, and this is continued until the merged model converges to a stable solution. The model used for the first iteration consists of random real numbers representing Bragg spot intensities. In this way the number of correlations is greatly reduced from *N* ^*n*^ (where each pattern is compared with all others) to 〈*m*〉*Nn* (through our use of a cumulative model), for *N* patterns and *n* indexing possibilities, where 〈*m*〉 is the expected number of iterations. This is the real power of the EM algorithm and it results from the use of a model where the output from one iteration is fed back to the following iteration to improve the outcome. For the case of space group *P*6_3_, there are two indexing modes ({*h*, *k*, *l*} and {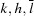}) that cannot be distinguished without considering the full reflection intensity information. The algorithm is outlined in the form of pseudo­code in the scheme below and as a flow chart in Fig. 1 ▶.[Chem scheme1] Using this algorithm, we aim to solve the following optimization problem
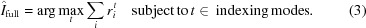
As for the Monte Carlo integration method (Kirian *et al.*, 2010 ▶), the intensities from the diffraction patterns are merged into the diffraction volume by averaging multiple measurements of the same Bragg spots, and in this way partiality is dealt with. This means a larger number of measurements (or diffraction patterns) should lead to a more accurate approximation to the real model of full reflections, with the error reducing as the inverse square root of the number of patterns.
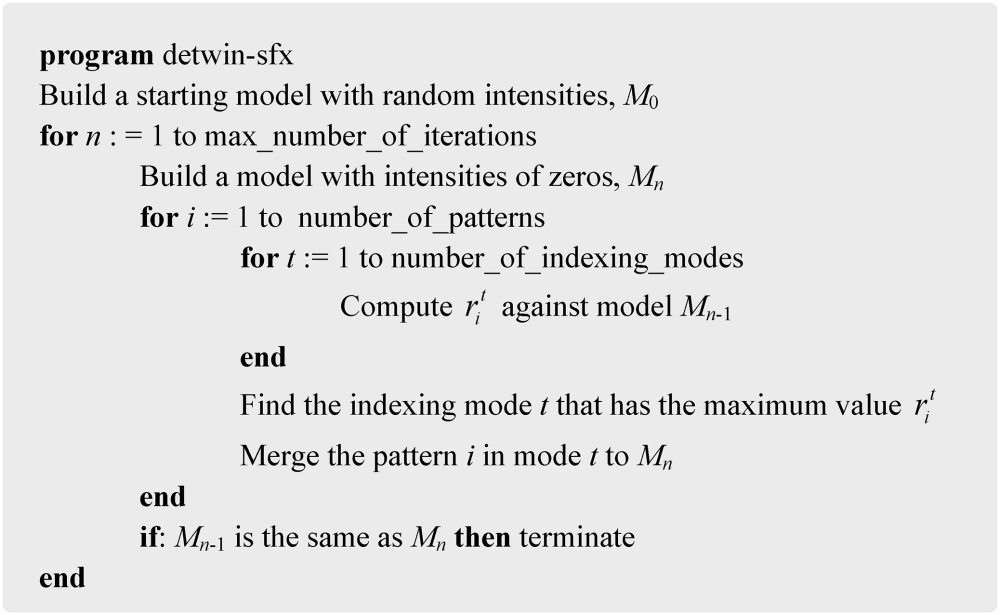


### 2.3. Error metrics   

For simulated data, the full reflection model is known and can be used as a reference to check whether the detwinning is successful. It is straightforward to compute the correlation between the detwinned model and the reference model. For the purposes of clarity, we label the indexing mode that is consistent with the reference model as the consistent indexing mode (CIM); the other indexing modes are labelled alternative indexing modes (AIM). For the case where there are multiple AIMs, a subscript can be used, such as AIM_1_, AIM_2_ *etc*. For the *P*6_3_ space group there is only one AIM, so we label the two indexing modes as CIM and AIM when comparing merged models with the reference model. Because the model indexed in one mode can be mapped to the other modes, the CIM and AIMs are interchangeable. To facilitate the following discussion, the indexing mode for the target model that yields the best agreement with the reference model is labelled as the CIM. These reference intensities from the known structure and its indexing mode are used only for comparison purposes to monitor errors in the analysis of experimental data, and they are never used as inputs to guide the detwinning at any stage.

For experimental data, we used Photosystem I (PSI) data collected during SFX experiments at the Linac Coherent Light Source (LCLS; Stanford, California, USA). The atomic co­ordinates of PSI [PDB (Berman *et al.*, 2000 ▶) code 1jb0; Jordan *et al.*, 2001 ▶] were used to compute the theoretical model reflection intensities, which were then used as a reference model to evaluate detwinning accuracy.

The model building process was monitored using a target score, defined as
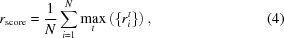
where *N* is the number of patterns. This score will increase as the merged model is improved on, by incorporating more diffraction patterns into the merged model in a consistent manner. This target score will reach a maximum when all the patterns are correctly indexed.

## 3. Results   

### 3.1. Performance evaluation using experimental data   

**Figure 2:**
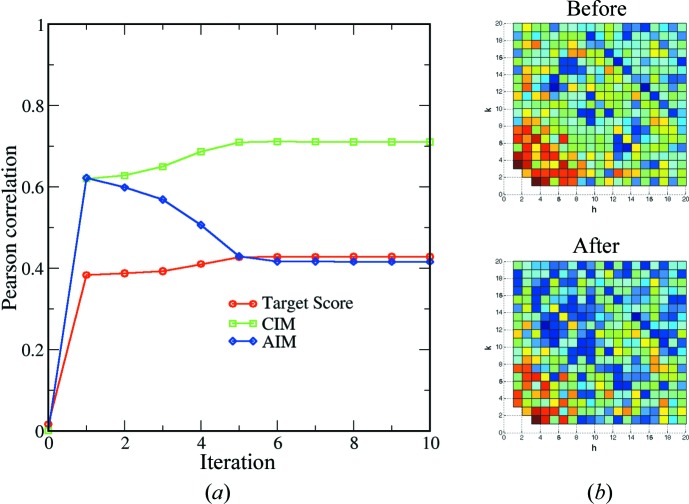
Test results of the detwinning algorithm with an experimental data set collected at LCLS for photosystem I. (*a*) The detwinning process. The reference intensity is the theoretical value calculated from the PDB model (pdb code 1jb0). The blue and green curves are the correlation coefficients between the theoretical and merged intensities at two indexing modes. The red curve is the target score, *i.e.* the average of the highest correlation coefficients between each pattern and the merged model. (*b*) The intensity distribution within the first quadrant in the plane of *l* = 0 in reciprocal space. The symmetry of the twinned data (top) disappears after applying the EM detwinning algorithm (bottom).

Photosystem I (PSI) belongs to space group *P*6_3_, which has an indexing ambiguity of {*h*, *k*, *l*} and {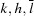}, so that there are two ways to merge any two diffraction patterns. A data set for PSI consisting of 17 106 indexed patterns has been collected at LCLS. After auto-indexing analysis, the unit-cell parameters were identified and found to be consistent with the model solved using synchrotron data from macrocrystals (PDB code 1jb0). Our EM algorithm was applied to this data set and the output model compared with the theoretical form factors calculated from the known atomic model (PDB code 1jb0). The detwinning process is summarized in Fig. 2 ▶. The merged model at each iteration was compared with the reference model (theoretical form factors of the PDB structure) at two indexing modes (the consistent and alternative indexing modes, CIM and AIM). After six iterations, the merged model converged to a stable solution. Fig. 2 ▶(*a*) shows that the initial correlations are nearly zero because the starting model consists of random positive numbers (hence no correlations are expected). The correlations between the output model from the first iteration and the reference model (1jb0 protein intensity) reached about 0.62 for both indexing modes. The orientations of the 17 106 patterns used in this test are classified into two groups of about the same size after the first iteration. After the second iteration, the correlation coefficients between the merged model and the reference model started to diverge and stabilized at 0.71 and 0.41 for the two indexing modes. The monitoring score [equation (4)] also increased to about 0.42, as shown by the red curve in Fig. 2 ▶(*a*). Because the patterns were obtained experimentally, the actual orientations were unknown beforehand, so it is difficult to evaluate the number of patterns that have their orientations recovered correctly. Nevertheless, using the theoretical intensity as a reference, a most likely indexing mode for each pattern can be assigned based on the correlations described in the *Methods*, §2.1, equation (2). Our EM algorithm recovered 94.6% of the diffraction patterns (16 188 out of 17 106) in a manner consistent with the indexing modes assigned using the theoretical model as the reference. In Fig. 2 ▶(*b*), an intensity slice through the plane *l* = 0 in reciprocal space is used to demonstrate the differences between the merged intensities before and after detwinning. For clarity, only the first quadrant with *h* < 20, *k* < 20 is shown. As mentioned, for the *P*6_3_ space group the indexing ambiguity occurs for {*h*, *k*, *l*} and {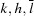}. In the plane *l* = 0, a symmetric distribution is expected for the twinned intensity, because the Miller indices {*h*, *k*, 0} and {*k*, *h*, 0} are equivalent before resolving the indexing ambiguity. This symmetry is clearly observed in the merged data from the original experimental data set (top of Fig. 2 ▶ *b*), while the bottom of Fig. 2 ▶(*b*) shows the result of the detwinning, showing that the artificial symmetry due to twinning effects is not present after applying our EM detwinning algorithm.

### 3.2. Performance evaluation using simulated data   

**Figure 3:**
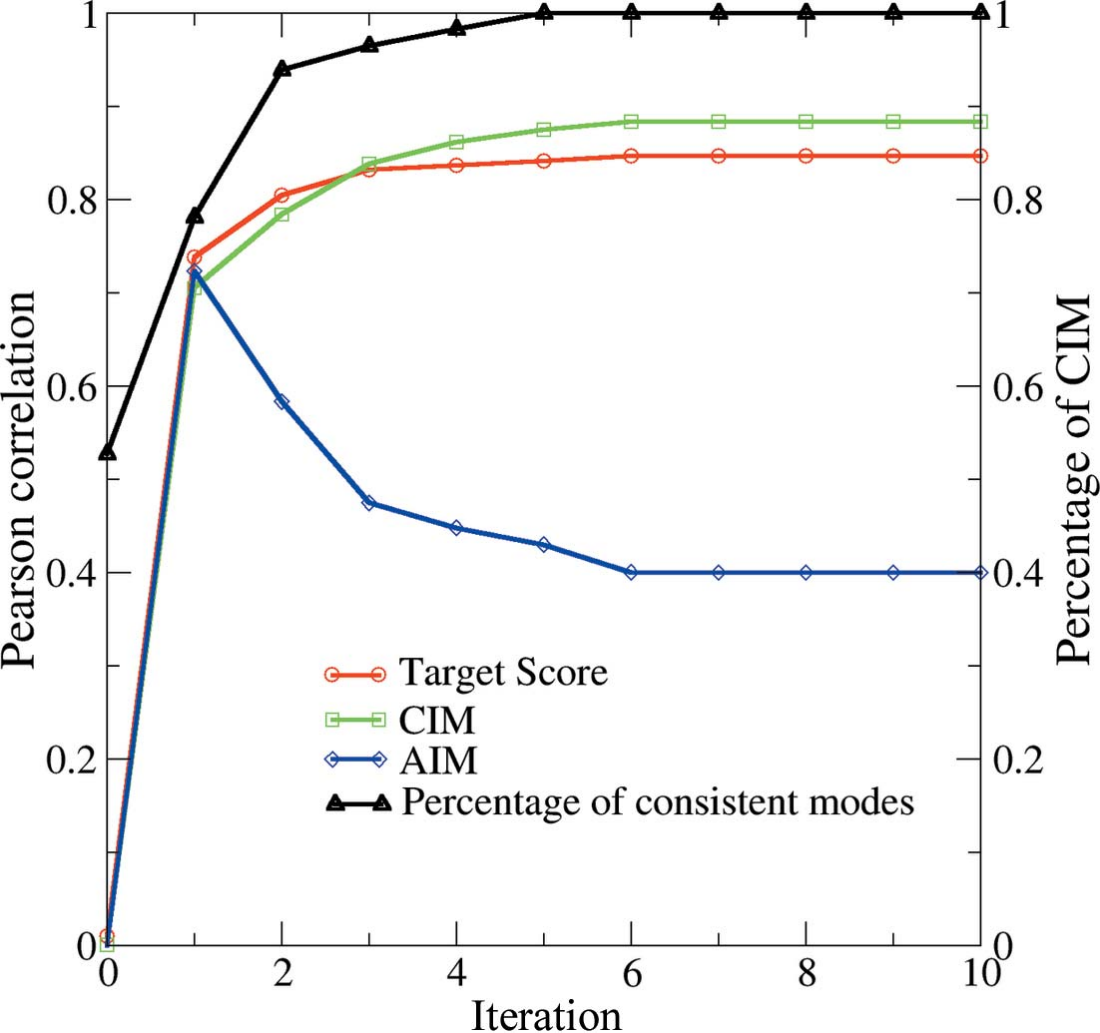
Summary of full intensity reconstruction and the progress of detwinning for the simulated data set. The indexing ambiguity breaks after six iterations, and almost all patterns are indexed consistently (line with triangles). All the other annotations are the same as in Fig. 2 ▶(*a*).

To quantify the performance of the algorithm further, two types of simulation were carried out. The first type of simulation was conducted following the same approach as that of Brehm & Diederichs (2014 ▶). The theoretical intensities were computed using the *sfall* program in the *CCP4 package* (Winn *et al.*, 2011 ▶), based on PSI in space group *P*6_3_ with unit-cell parameters from the PDB model (PDB code 1jb0). The simulated patterns were based on experimental data collected at the LCLS, as described in the previous section. For each pattern, the Bragg peaks were re-indexed following either indexing mode with equal probability. The experimental intensities were replaced with the calculated values, and the partial reflections were modelled using partialities drawn randomly from a uniform distribution (from 0 to 1). Shot noises, following the Poisson distribution, were added to the simulated intensities. For the simulated data set, where information about the indexing mode for each diffraction pattern is known, the performance of the algorithm can be evaluated directly by counting the number of correctly recovered indexing modes. The results from a typical test run are summarized in Fig. 3 ▶, showing that the vast majority of the patterns have been indexed consistently with respect to the reference model.

The detwinning process is similar for the case of the experimental data set discussed in §3.1. The starting model was not correlated with the reference model in either indexing mode, as indicated by a zero correlation coefficient. Then, after the first iteration, the patterns fall into two classes based on the likelihood of being in each indexing mode and half of the patterns are correctly assigned in this iteration, as expected. As the iteration continues, the indexing ambiguity breaks down, reflected by the growing percentage of correctly recovered indexing modes. The target score increases and reaches a stable value of about 0.85 after a few iterations. The correlation between the merged model and the reference model (*i.e.* the theoretical intensity calculated from 1jb0) is also plotted in Fig. 3 ▶. The merged model is compared with the reference model at the two indexing modes for space group *P*6_3_. As the detwinning progresses, the information embedded in the diffraction patterns is incorporated into the merged model, the correlation of which with the reference model increases to 0.71 for the CIM and 0.69 for the AIM after the first iteration (Fig. 3 ▶). This initial small propensity towards one indexing mode over the other leads to an eventual breakdown of the indexing ambiguity. We note that the correlation coefficient between the reference and merged models increases to 0.88 after six iterations in the CIM, while the coefficient for the model in the AIM gradually reduces to about 0.40.

**Figure 4:**
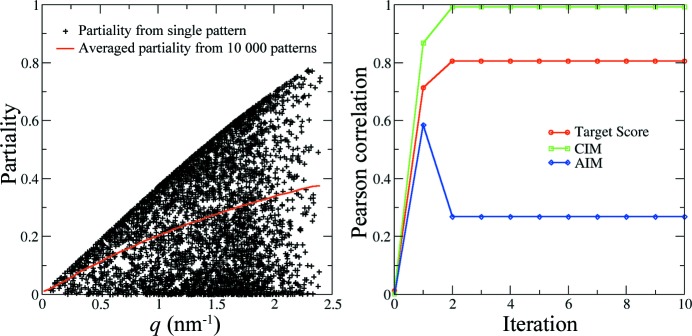
The detwinning process for a simulated data set, with the emphasis on systematic modelling of partiality. (*a*) The relation between partiality and resolution. At higher resolution (larger *q* values), the recorded intensity is more accurate and better represents the corresponding full reflection. (*b*) Within two iterations, the indexing ambiguity problem is solved and almost all patterns are indexed in consistent modes.

The partiality of intensity is not randomly distributed but depends on many factors, including crystal size, orientation and mosaicity and the monochromaticity of the radiation. Here, we have simplified the problem by assuming that the crystal sizes are about the same, which is achievable using experimental crystal size selection, or data screening. For the second type of simulated dataset, the partiality was modelled using the program *partial_sim* in the *CrystFEL* suite (White *et al.*, 2012 ▶). Fig. 4 ▶(*a*) shows the relationship between partiality and resolution. Because the Ewald shell is thicker at higher resolution, the larger overlaps between the Ewald shell and the Bragg spots result in better intensity measurement, reflected in larger partialities at higher scattering angles. The performance of the algorithm for a data set of 10 000 patterns compiled using this approach is summarized in Fig. 4 ▶(*b*). In that case, the model intensity reconstruction converges faster, and the indexing ambiguity is resolved after just two iterations. The final correlation coefficient compared with the theoretical intensity is about 0.99, with only two patterns (out of 10 000) indexed incorrectly.

### 3.3. Consistency and robustness of the algorithm   

**Figure 5:**
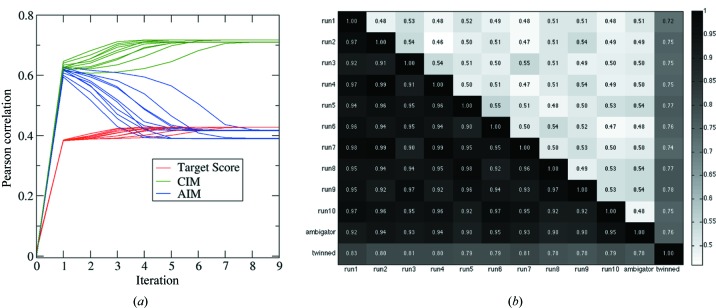
The EM algorithm results are consistent. (*a*) The program was run ten times with randomly generated starting models, and the final output models have nearly the same correlations compared with the theoretical models. (*b*) Pairwise comparison of the models from ten runs, the results from the program *ambigator* in *CrystFEL* and the twinned data. The lower triangular matrix shows the correlations when compared in CIMs and the upper triangular matrix shows the correlations in AIMs. Note that the correlations are very similar for twinned data in these two modes (dark-grey colour, last row and last column) while the other models are very consistent, giving correlations larger than 0.90 when indexed in the same modes.

The detwinning results are consistent with each other. For the same set of experimental data described in §3.1, the EM algorithm was applied ten times with different randomly generated starting models. The ten detwinning processes are shown in Fig. 5 ▶(*a*), indicating the convergence of detwinning within ten iterations. Although the convergence speeds vary, the final results are consistent: (i) the correlations with the reference model computed from the PDB structure are very similar in both indexing modes; (ii) the target scores converge to almost the same value. The consistency between the final models from the ten runs was also evaluated by computing pairwise correlations. It is also worth comparing the EM detwinning results with those from the method implemented in the *CrystFEL* package, the program *ambigator*, which utilizes the average values of the correlations between one pattern and an ensemble composed of other patterns. We also included the merged intensities without detwinning operations in the pairwise comparisons. For these comparisons, the correlations were computed for the subset of reflections within resolution shells between 3 and 5 Å. This subset of reflections contains about 55% of the total reflections (111 452 out of 204 092), well representing the reflections in reciprocal space and reducing the influence of extremely large intensities at low resolutions. The results are summarized in Fig. 5 ▶(*b*), where the correlation between each model pair is computed twice, considering the two indexing modes: one for the CIM and the other for the AIM. The lower triangular matrix shows the correlations between the models when they are indexed in the same modes (CIM), and the upper triangular matrix contains the correlations between models that are in alternative indexing modes (AIM). The heat map shows that the ten models, when indexed in the same modes, have very large correlations (>0.90), indicating good self-consistency. On the other hand, the correlations between models are smaller than 0.55 when they are indexed in different modes. The results from the *ambigator* program are also strongly correlated with all ten models obtained from the EM algorithm, with correlations from 0.90 to 0.95. By contrast, the twinned intensities are not strongly correlated with the detwinned results. Based on the correlations between the twinned model and all other detwinned results, the two indexing modes are barely distinguishable for the twinned model (with correlations between 0.70 and 0.80), as shown in the heat map (Fig. 5 ▶ *b*), where the last row and the last column correspond to correlations between detwinned results and the twinned data for the two indexing modes.

**Figure 6:**
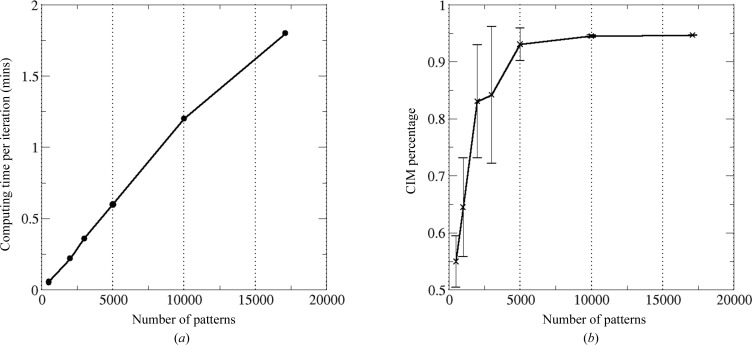
The speed of the EM algorithm and the requirements on the data set. (*a*) The time required for a single iteration is linearly dependent on the number of diffraction patterns. (*b*) For the case of PSI experimental data, a minimum set of 5000 diffraction patterns is required to guarantee reliable converged models. The CIM percentage shown on the vertical axis means the percentage of recovered indexing modes that are consistent with the reference model. When the sample size is smaller than 500, the EM algorithm fails to resolve the indexing ambiguity. For the cases where 1000 to 3000 patterns form the test set, detwinning can be successful but the large error bars suggest that the success rate is not guaranteed, so more runs would be desired.

The EM algorithm is fast and has the advantage of linear scaling with respect to the number of patterns. It took about 18 min to process the experimental data set composed of over 17 000 patterns on a MacBook Pro with a 2.8 GHz Intel Core i7 two-core processor. The computation time is plotted as a function of the number of patterns in Fig. 6 ▶(*a*), showing a linear dependence relation. The current implementation of the algorithm utilizes a single computing core, and detwinning can be speeded up by parallelizing the correlation calculations that can be distributed to many computing cores when a larger dataset needs to be processed.

The EM algorithm has also been evaluated using smaller data sets and the results suggest that the algorithm is very robust. From an experimental data set composed of over 17 000 diffraction patterns of PSI crystals, subsets of the patterns were randomly selected to form data sets of 500, 1000, 2000, 3000 and 10 000 patterns. The percentage of consistently indexed patterns is summarized in Fig. 6 ▶(*b*). When the data set was composed of more than 5000 diffraction patterns, we found that more than about 90% of the patterns were indexed consistently. When the data set was composed of a smaller number of patterns, the algorithm became less reliable, indicated by the smaller percentage and larger fluctuations. This may be because the merged intensities could not accurately approximate the full reflections if the data set were not large enough. This can be understood from the fundamental properties of Monte Carlo integration, which requires sufficient sampling of each Bragg spot. Another extreme case is that the consistently indexed patterns are close to 50% (failed in detwinning) when the number of patterns in the test data set is too small (500 in this test case), as shown in Fig. 6 ▶(*b*).

## 4. Discussion and conclusions   

We have demonstrated that the expectation maximization (EM) algorithm can be applied to solve the indexing ambiguity problem which arises in serial femtosecond crystallography (SFX) and other X-ray diffraction experiments where ‘still’ reflections are collected without a goniometer, such as the use of a lipid cubic phase nanocrystal injector at a synchrotron. In SFX, the experimental diffraction pattern from a nanocrystal depends on several factors, including crystal orientation, size, mosaicity and shape, the X-ray beam profile, and the interaction region between the crystal and the X-ray pulse (for example, crystals may be only partially illuminated by a highly focused beam). Submicrometre-sized crystals can result in shape-transform effects if the coherent beam is wider than the crystal. The effect of these will be investigated in a subsequent paper devoted to the smallest crystals. The X-ray beam profile varies from shot to shot, but seeding methods can improve the beam quality. In the original Monte Carlo sampling approach for nanocrystals at an XFEL (Kirian *et al.*, 2010 ▶), it was expected that this approach would average over all stochastic experimental variables, and this behaviour has been confirmed by the *N* ^−1/2^ fall-off in the error of structure factor measurement with the number *N* of diffraction patterns. It has been shown that the pairwise correlation between diffraction patterns can be used to break the indexing ambiguity. Although the correlation function calculated from partial intensities may not be highly accurate, the information is sufficient to identify the correct orientation from incorrectly indexed twins. In such operations, qualitative results are critical. As long as the correct indexing mode yields slightly higher correlations compared with incorrect indexing modes, then the correlation-based algorithm should work. This would explain the success of the method for distinguishing the correct indexing mode from its twin in an experimental data set where *r*_score_ is less than 1.0 (*r*_score_ = 1.0 only in the case where full reflections are recorded in each pattern).

The partiality of reflection intensity does not prevent recovery of the correct indexing mode. If the patterns are randomly indexed in two possible twin-related modes for photosystem I crystals, the merged (twinned) full intensity will have a correlation coefficient of about 0.71 compared with the correct model (the theoretical values that were used to generate the patterns). After resolving the ambiguity, the merged model is almost identical to the reference model (the correlation coefficient is close to 1.0), even in the case where the average partiality is smaller than 0.4 (see Fig. 4 ▶ *a*).

**Figure 7:**
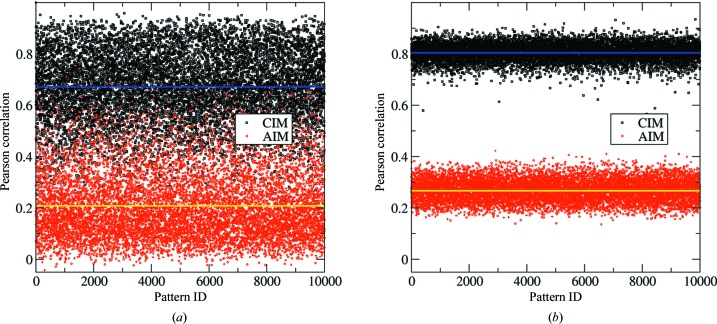
Correlations between pairwise SFX patterns, and correlations between SFX patterns and a model with full reflections. The data set is from simulations obtained using the *partial_sim* program (see Fig. 4 ▶). (*a*) Diffraction patterns are compared with a randomly selected pattern, and correlation coefficients for two indexing modes are calculated for every pair of patterns (black and red). The average coefficients for each indexing mode are shown with blue and yellow lines. (*b*) Correlations between the diffraction pattern and the merged intensities after three iterations. It is clear that the correlations shown in (*b*) separate the two indexing modes more reliably, even in the presence of severe partiality (<40%, see Fig. 4 ▶ *a*).

Using patterns simulated using the *partial_sim* program with the same parameters that were used to generate the data set in Fig. 4 ▶, we conducted a comparison between two measures: (i) using correlations between all patterns and a randomly selected pattern (*i.e.* each correlation coefficient is taken between two sets of partial reflections), and (ii) using correlations between patterns and full intensities (the correlation between partial and full reflections). The results shown in Fig. 7 ▶ indicate that our correlation method based on the EM approach is more reliable for distinguishing indexing modes. As the iteration progresses, the merged intensities move closer to the full intensities and the separation of two indexing modes becomes more pronounced, eventually moving to the state of Fig. 7 ▶(*b*).

Real experimental data often suffer from imperfect measurement, such as saturated pixels at very bright spots at low resolution, or low intensity at very high resolutions. Such imperfections introduce errors in the correlations. One strategy to reduce the influence of these saturated pixels and low-signal pixels is to exclude these data points from the correlation calculations. In the current implementation of the EM detwinning program, the very low-resolution data were ignored for correlation calculations, because extremely large values at low resolution will make the correlations less sensitive to the differences due to different indexing modes.

In summary, we have found that an algorithm based on the expectation maximization approach can effectively resolve the indexing ambiguity problem, which is unavoidable in serial femtosecond crystallography for many space groups. The method is implemented within the *CrystFEL* framework, and the source code is available upon request from the authors.
